# Analysis of hospital costs by morbidity group for patients with severe mental illness

**DOI:** 10.1080/07853890.2022.2048884

**Published:** 2022-03-23

**Authors:** Vicent Caballer-Tarazona, Antonio Zúñiga-Lagares, Francisco Reyes-Santias

**Affiliations:** a Finanzas Empresariales, Universitat de València, Valencia, Spain; b AMADEM, Alicante, Spain; c Master Gestión Sanitaria, UPV, Valencia, Spain; d IDIS, Santiago de Compostela, Spain

**Keywords:** Mental Illness, length of stay, hospital costs, comorbidities

## Abstract

**Objectives:**

The goal of this study is to analyse hospital costs and length of stay of patients admitted to psychiatric units in hospitals in a European region of the Mediterranean Arc. The aim is to identify the effects of comorbidities and other variables in order to create an explanatory cost model.

**Methods:**

In order to carry out the study, the Ministry of Health was asked to provide data on access to the mental health facilities of all hospitals in the region. Among other questions, this database identifies the most important diagnostic variables related to admission, like comorbidities, age and gender. The method used, based on the Manning–Mullahy algorithm, was linear regression. The results were measured by the statistical significance of the independent variables to determine which of them were valid to explain the cost of hospitalization.

**Results:**

Psychiatric inpatients can be divided into three main groups (psychotic, organic and neurotic), which have statistically significant differences in costs. The independent variables that were statistically significant (*p* <.05) and their respective beta and confidence intervals were: psychotic group (19,833.0 ± 317.3), organic group (9,878.4 ± 276.6), neurotic group (11,060.1 ± 287.6), circulatory system diseases (19,170 ± 517.6), injuries and poisoning (21,101.6 ± 738.7), substance abuse (20,580.6 ± 514, 6) and readmission (19,150.9 ± 555.4).

**Conclusions:**

Unlike most health services, access to psychiatric facilities does not correlate with comorbidities due to the specific nature of this specialization. Patients admitted to psychosis had higher costs and a higher number of average stays

**KEY MESSAGES**
- The highest average hospital expenditure occurred in patients admitted for psychotic disorders.
- Due to the particularities of psychiatry units and unlike other medical specialties, the number of comorbidities did not influence the number of hospital stays or hospital expenditure.
- Apart from the main diagnostic group, the variables that were useful to explain hospital expenditure were the presence of poisoning and injuries as comorbidity, diseases of circulatory system as comorbidity, history of substance abuse and readmission.

## 1. Introduction

Currently, it is of vital importance to care for patients with multiple pathologies by addressing the treatment of single and independent diseases, as this leads to greater difficulties in clinical management [1,2]. Furthermore, various authors suggest transforming a system focussed on healing the sick for another that cares for and treats the chronically ill with multiple pathologies, placing the emphasis on the importance of prevention and health maintenance. At present, the model we use was not conceived of for sustaining the treatment and care of such an ageing population with many problems of accumulative and comorbid diseases [3,4]. In the case of psychiatric admissions, there is also evidence that a greater medical comorbidity implies greater cost and hospital stays [5].

However, at the present moment, there is a lack of information regarding the interrelation of diseases in clinical practice, largely due to the exclusion from clinical works of those patients with one or more disorders or those of advanced age, factors which should be borne in mind for future research [6].

One of the main problems with patients with various chronic diseases is the interaction of prescribed medicines, which can have serious effects, both on the health of the patient and the economic cost [7].

In this context, identifying and classifying patients with various pathologies and high costs is a priority in providing better treatment of psychiatric patients [8].

The goal of this study is to identify which variables offer an explanation for hospital expenditure in the case of psychiatric admissions and to establish an econometric predictive model using different techniques.

## 2. Method

### 2.1. Study design and data source

#### 2.2.1. Design

Observational, transversal and retrospective study of all psychiatric admissions to hospitals in the Valencia Community in 2015.

#### 2.1.2. Subjects

All patients admitted in the different hospitals of the Valencia Community in 2015. The dataset taken from the files of the Ministry of Health MBDS was of 6705 patients and 7,752 admissions (which all psychiatric admissions records for all adults hospitalised in Valencia in 2015). From the Valencian public health service, psychiatric hospital care is offered in two types of centres: short-stay psychiatric units in general hospitals and medium-stay monographic mental health hospitals. All admissions in these two types of centres were considered in this study. In addition, there are other private psychiatric care centres but for which the information was not available.

The diagnosis-related groups (DRGs) are drawn up at the time of discharge from hospital in the admissions department. Only one DRG is applied to each patient discharged. This means that, if a patient who has been admitted to hospital for a psychiatric pathology is transferred to another clinical service, as a consequence of a non-psychiatric comorbidity, the information on the consumption of resources of that patient for the psychiatric area will be lost, leaving only the information on the consumption of resources in the clinical service where he/she has been discharged.

The nine groups of psychiatric illnesses were obtained from the principle diagnosis on admission and were dementias and other diagnostic disorders, schizophrenia and affective psychoses, other neuroses excepting depressive, depressive neuroses, personality disorders, childhood and adolescent disorders, non-specified mental disorders and other diagnoses. Moreover, psychiatric diagnoses were grouped into three groups whose differences in expenditure are statistically significant. These are psychotic group (psychotic disorders); organic group (dementias and other organic disorders, adjustment disorders and non-specified mental disorders); and neurotic group (other neuroses, dysthymias, personality disorders and childhood and adolescent disorders).

For the medical comorbidity, the presence of any pathology included in each of the main chapters of ICD-9 was taken into account. Variables that were not statistically significant were withdrawn from the model until the system of regression was found in which all the variables were statistically significant.

### 2.2. Patient and public involvement

The data used were obtained from the electronic database of the Spanish Ministry of Health. This anonymous database consisted of reason for admission, length of stay, age, sex and associated ICD diagnoses.

All admissions in the psychiatric units of the hospitals of the Valencian Community were required

### 2.3. Hospital cost analysis

First, the differences in average stay and expenditure were evaluated for the nine psychiatric diagnoses groupings, largely coinciding with the psychiatric DRGs, for a sample of psychiatric admissions of the Valencia Community hospitals corresponding to 2015 from the Spanish Health Ministry’s MBDS database. Second, we verified the influence of certain variables, such as age, sex, the presence of personality disorders, the presence or lack of readmission, burden of medical morbidity, substance abuse and total burden of psychiatric illness on the aforementioned average stay and expenditure.

### 2.4. Statistical methods

First, the statistically significant variables were identified and an explanatory model of hospital expenditure designed.

The skewed distribution of health expenditure data is one of the great challenges that health economists face. The most common statistical methods used to handle this type of data are log models, ordinary least-squares on ln (y) and generalized linear models.

The Manning–Mullahy log [9] was used to determine the most appropriate method depending on the shape of data distribution measured by kurtosis, that is a coefficient which indicates whether the distribution shape is normal, flatter or thinner (peaked).

As the coefficient of kurtosis was greater than 3, linear regression using least squares was the most indicated statistical technique.

The dependent variable of the model was the log of healthcare expenditure. Transformation to a log was carried out, as in this way the distribution of healthcare expenditure showed a better fit, for which the application of the Smearing Estimator was required [10,11]. In order to apply linear regression, in a data set that does not present a normal distribution, a logarithmic transformation of this data set is required. The result of the regression is presented therefore in logarithmic terms. When these values are retransformed from the logarithm to obtain them in terms of real units of the observed variable, precision and goodness of fits are lost.

The Smearing Estimator is a coefficient that corrects these inconveniences so that the resulting value of the regression is closer to the real value of the observations.

The independent variables were age and sex of the patient, diagnostic grouping in nine groups of psychiatric illnesses, presence of substance abuse, readmission (In the same year 2015 or with readmission history prior to that year) and the medical morbidity burden grouped by ICD codes and chapters.

## 3. Results

The descriptive analysis of the sample studied (*N* = 7.752) shows a distribution with 50.2% of male subjects and slightly less for female (49.8%) typing error and an average age of 47 (SD 20.38). Hospital stays were on average 12.97 d and the average cost per patient €9,288.6. In the sample, 52.6% of the patients presented diagnoses of psychotic disorders (schizophrenia and affective psychoses, DRG 430), 14.2% other neurotic disorders including mild depression (except dysthymias, DRG 427), 11.9% organic mental disorders including dementias (DRG 429) and lastly personality disorders and impulsivity including eating disorders (DRG 428). The most relevant information on each diagnostic group is shown in Table 1.

**Table 1.** Patient characteristics and mean annual hospital cost by sector and total.

	Number of admissions (%)	Hospital cost^a^		*p* Value	Hospital stays	
		Mean	SD		Mean	SD
Overall	7752	9266.00	6037.60		1.9	17.5
Age				**.000**		
0–18	567 (7.3)	8399.80	5125.10		12.5	14.5
19–24	524 (6.8)	9345.20	5464.40		12.8	15.4
25–34	1069 (13.8)	9617.70	4466.50		12.8	12.1
35–44	1609 (20.8)	9611.50	5529.40		13.3	15.9
45–54	1527 (19.7)	9800.60	6181.40		13.7	18.3
55–64	853 (11.0)	10,278.20	8592.90		15.2	26.4
65+	1603 (20.7)	7917.30	5880.10		10.7	16.8
**Sex**				**.000**		
Male	3888 (50.2)	9533.00	6555.00		13.3	19.4
Female	3864 (49.8)	8997.00	5455.00		12.5	15.4
**Diagnostic grouping**				**.000**		
1. Dementias and other organic mental disorders	925 (11.9)	5908.80	3585.70		8.4	10.9
2. Psychotic disorders (schizophrenia and affective psychosis)	4075 (52.6)	11,753.20	6579.00		16.5	21.1
3. Other neuroses, except depressive, anxiety disorder, sleep disorder, Sexuality Disorder	1100 (14.2)	7370.20	4592.90		9.9	12.4
4. Depressive neuroses (dysthymias)	165 (2.1)	6389.00	2229.70		7.2	7.2
5. Personality disorders and impulsivity (eating disorder)	910 (11.7)	6158.50	3240.00		9.1	10.4
6. Adaptive disorders and V codes	311 (4.0)	5966.20	2258.60		6.8	6.9
7. Childhood and adolescence disorders	157 (2.0)	6902.60	4507.10		9	13.2
8. Unspecified mental disorders	68 (0.9)	5903.00	3002.30		7	9.5
9. Surgical intervention with main diagnosis of mental disorder	41 (0.5)	8878.10	4027.00		11.5	9,7
Groups of expenditures				**.000**		
Psychotic group	4075 (52.6)	11,753.18	6579.01		16.53	21.5
Organic group	1304 (16.8)	5922.19	3285.63		7.95	10.03
Neurotic group	2373 (30.6	6832.41	4018.02		9.38	11.41
Number of psychiatric comorbidities				**.575**		
1	5952 (76.8)	9290.36	6273.18		12.91	18.32
2	1126 (14.5)	9276.80	5060.05		12.98	14.21
3	674 (8.7)	9032.83	5383.70		12.59	15.07
Number of total comorbidities (for the same patient)				**.003**		
0	1183 (15.3)	9478.00	6539.40		13.2	19.1
1	1317 (17.0)	9660.50	5964.70		14	17.3
2	1198 (15.5)	9512.60	5813.60		13.2	16.8
3	1059 (13.7)	9257.60	4845.80		12.7	13.3
4	814 (10.5)	8981.90	4364.30		11.8	11.7
5	590 (7.9)	8927.80	4405.20		12.1	11.6
6 or more	1591 (20.5)	8872.50	6836.80		12.5	19.9
Abuse of substances				**.367**		
No	5334 (68.8)	9250.40	6558.50		13.1	19.2
Yes	2418 (31.1)	9300.40	4689.10		12.4	12.9
Admission				**.031**		
N° First admissions	6705 (86.0)	9316.60	6045.40		13	17.5
Nª Readmissions	1047 (14.0)	8942.20	5979.50		12.1	17.3
Personality disorders				**.000**		
No	6907 (89.1)	9459.80	6142.90		13.1	17.9
Yes	845(10.9)	7682.30	4807.20		11	13.4

^a^Expressed in euros.

Regarding age, the group of between 25 and 65 years old constituted 65.2% of total admissions, while those over 65 at 20.7% and under 25 s at 14.1%.

In order to reduce the number of diagnostic groups to simplify the calculation of the regression, these can be grouped into three categories of different diseases: (1) psychotic disorders, (2) dementias and other organic disorders, adjustment disorders and other unspecified mental disorders and (3) other neuroses, dysthymias, personality disorders and childhood and adolescent disorders. The average expenditures for these three groups respectively are 11,796.2, 6796.4 and 5925 euros, which would correspond to a “psychotic group”, an “organic group” and a “neurotic group”.

Of the total of registered admissions, 31.1% presented substance abuse, a factor that entailed a lower number of average stays but not statistically significant. Evaluating the burden of psychiatric illness according to the number diagnosed in each case analysed, 76.8% of the sample had only one psychiatric diagnosis, 14.5% two diagnoses and 8.7% three or more diagnoses. Nevertheless, no relevant differences were appreciated either in the number of average stays or in hospital expenditure. Neither gender nor age was statistically significant factors when explaining hospital expenditure during admission or hospital stays. The 14% of admissions are from re-admitted patients, of which 26.14% had a clinical history of substance abuse, which is not very different from the percentage of admissions (31.1%) with a history of substance abuse for all admissions. Patients with a diagnosis of psychosis (50.85%) were the most frequent among those readmitted.

Regarding medical comorbidities, the most frequent are hypertension (18.3%), smoking^1^ (13.6%), prolonged use of other pharmaceuticals (11.6%), diabetes (10.8%) and metabolic disorders (6.7%) (Table 2).

**Table 2.** Principal comorbidities by ICD-9 chapter.

Comorbidities	*N*	%
Hypertension	1422	18.3
No comorbidity	1183	15.3
Smoking	1053	13.6
Infectious intestinal disease	960	12.4
Diabetes	840	10.8
Confusional state (Delirum^2^)	718	9.3
Other disorders of consciousness	594	7.7
Metabolic disorders	516	6.7
Atrial fibrillation	465	6
Dysphagias	462	6

By chapter or disease groups according to IDC-9, the most frequent morbidities were diseases of the nervous system and senses (52.3%), then mental, behavioural and neurodevelopmental disorders (52.3%) and thirdly, classification of factors that influence the state of health (V codes) (45.3%).

With respect to the section on medical comorbidities and their relation to the different groups of psychiatric disorders analysed, it is worth noting the statistically significant correlation between neoplasms and circulatory system disorders in diagnostic grouping 1 (dementias and other organic mental disorders), as with that of addictive behaviours and diagnostic grouping 5 (personality disorders and impulsivity).

Mental comorbidities were not found to have a statistically significant influence on higher healthcare spending, only the main diagnosis of mental illness (see Annexe 1).

Moreover and related to the number of physical multi-morbidity in the sample and its possible impact on expenditure, only one comorbidity was statistically significant.

Table 3 shows the regression model obtained after discarding those variables that were not statistically significant and whose adjusted R squared was 41.1%.

**Table 3.** Association of patient characteristics with hospital cost through regression model.

		Confidence interval		
	B	Max	Min	Sig.
Psychotic group (Constant)	9.31	9.31	9.30	0.00
Organic group	−0.69	−0.68	−0.71	0.00
Neurotic group	−0.58	−0.57	−0.59	0.00
Comorbidity presence (diseases of the circulatory system)	−0.03	−0.02	−0.04	0.01
Comorbidity presence (injuries and poisonings)	0.06	0.08	0.04	0.01
Readmission	−0.03	−0.02	−0.05	0.00

The three grouped diagnostic groups (“psychotic group”(constant), “organic group” and “neurotic group”) were statistically significant. Readmission also represented a lower cost and lower number of stays.

Regarding the section of medical comorbidities, only diseases of the circulatory system and injury and poisoning were statistically significant in explaining the model.

The remaining comorbidities, either gauged by the IDC-9 code or chapter and by explanatory variables were not valid in explaining the variability in hospital expenditure in the psychiatric units of the Valencia Community.

Substance abuse, despite being statistically significant, was discarded as an explanatory variable since the type of substance was not specified, which could distort the results.

The information shown in Table 3 is in logarithmic terms, therefore, to interpret the results, you would have to apply the exponential to each variable and multiply it by the Smearing Estimator, which in this case is 1.08.

Thus, for example, according to the regression, a patient classified in the neurotic group, without readmission and without the presence of comorbidities of the circulatory system or lesions or poisonings would have an estimated expenditure of = Exp (9.31) * 1.08 = 11,901.68 €.

In another example, a patient from the organic group, with a history of injuries and poisonings and with readmission, would have an estimated cost of = (Exp (9.31-0.69 + 0.06-0.03)* 1.08 = 6,124,35 €.

## 4. Discussion

Unlike with the majority of medical specialities, the length and cost of psychiatric admissions are not influenced by either by the age or number of comorbidities the patient presents. This is due to the greatest hospital expenditure being generated by schizophrenic patients, who do not have a very high average age and do not present as many medical comorbidities as other kinds of psychiatric patients, such as those with dementias or depressive neuroses. In general terms, psychiatric unit stays of elderly patients or a those with a high number of medical comorbidities are usually referred to other units or discharged for care by other out-of-hospital services.

In the UK, in a similar study, the results obtained indicated that the greatest expenditure was incurred by schizophrenic patients. Nevertheless, sex and other socio-demographic variables did prove to be statistically significant in explaining hospital expenditure [12]. On the other hand, the psychopathology, as is also concluded in this present article, was not useful in predicting the length and cost of psychiatric hospital admissions according to a study by Warnke et al. [13]

Two other studies [14,15] agree in indicating admission for psychosis as one of the main factors in explaining cost and length of stays, although the latter also indicates a statistical significance regarding the sex of the patient and the size of the hospital. However, in the systematic review, it is factors associated with depression that are closest related to a longer stay [16].

A study carried out in Canada and focussing exclusively on schizophrenic patients, found, as in the present study, that the presence of substance abuse meant a lower number of stays in psychiatric units as these patients are normally discharged after the intoxication [17].

According to Newman et al. [12], the factors associated with the length of the stay in psychiatric hospital services in London, UK were the following: diagnosis of psychosis, involuntary commitment, seriousness of disease, receiving ECT (electroconvulsive therapy), being unemployed, unstable housing or homeless and being female. Regarding the distribution of the reason for hospitalisation by diagnostic groups according to ICD-10, the most numerous was psychosis (F20–29) with 43.85%, followed by affective disorders (F30–39) with 24.74%, personality disorders (F60–69) with 8.44% and disorders attributed to substance abuse (F10–19) with 8.19%.

Adlington et al. [18] find that length and number of admissions are important indicators of quality of attention and are greater in the psychiatric wards for the elderly as a result of physical comorbidity and the need for attention. Along the same line Cheng et al. [19] identified factors associated with the length of stay, also finding a greater probability that psychiatric patients with longer stays in a large urban hospital were the elderly, with cognitive impairment, greater medical comorbidity and with episodes of agitation during the stay. Similar conclusions can be seen in the research of Bail et al. [20].

These studies contrast with the findings of our work, where psychiatric hospital stays of elderly patients characterised by a high prevalence of dementias are normally referred to other hospital services for chronic diseases and out-of-hospital care units.

A study with the goal of evaluating the prevalence and characteristics of physical diseases among psychiatric hospitalised patients [7] found that the socio-demographic variables of the sample were similar to previous studies [21]. In the aforementioned study, the principal psychiatric diagnosis group was schizophrenia together with schizotypal disorders and delusional disorders (F20–29) 45%, followed by mood disorders (F30–39) 33.3%, disorders from substance abuse (F10–19) 19.4%, neurotic disorders, disorders related to stress and somatoform disorders (F40–48) 16.1%. About 18.3% of the subjects were diagnosed with more than one psychiatric illness. In general terms, the results of the present work are in line with the study carried out in India.

In that same study, it was also found that 70% of patients had one or more associated physical disease, of which 38.3% had more than one, in accordance with the findings of Berren et al. [22], who concluded that 60% of people with mental illness develop serious medical comorbidities, as did Mohamad Isa et al. [23], who also found that 63.4% of the subjects in his study had comorbid physical diseases, among which were: metabolic (28.9%), endocrine (25.6%), haematological (18.3%), gastrointestinal (15%), cardiovascular (12.2%), neurological (9.4%) and stomatological disorders (8.3%).

Nevertheless, Singh et al. [21] found in their study that the principal physical disease was hypertension (29.1%) followed by repository diseases (15%), anaemia (12.5%), diabetes mellitus (10%), hepatic diseases (5.8%), ocular diseases (5%) and skin diseases (5%) [23]. They also reported that hyperlipidaemia (24.6%), hypertension (16.4%) and diabetes (12.7%) were the most common among the study population. In our study, we note in relation to the above, that 51.7% of the cases presented more than one medical pathology and 23% more than one psychiatric pathology.

The results of Sebestyen et al. [24] demonstrated that, of the comorbid psychiatric conditions researched, only dementia was related to an increase in length of stay. The degree of cognitive impairment shows a positive correlation with the length of stay and the cost of medical treatment. Given the high incident rate of affective syndromes, it may be supposed that comorbid depression increases the possibility of admittance to an internal medicine ward with somatic pain.

Although it is not the aim of this study, it can be noted that the Revolving Door phenomenon has become a public health issue, both for health and economic sides.

In this sense, we can point out that, regarding the revolving door phenomenon [25], our study does not show clear patterns that determine factors that explain a greater probability of readmission. This coincides with another study in Spain, in Aragon region, according to which psychiatric patients with readmissions account for 21.08% of all admissions to the Short Stay Psychiatric Unit under study [26].

These results contrast with other studies, such as that of Dias Neto and Nunes da Silva [27], which show that of the 3225 patients admitted in this period, 1276 (39.6%) were readmissions. Furthermore, a readmitted patient was admitted, on average, 2.6 times during the study timeframe. In Brazilian studies, Castro et al. found that 34% of hospitalizations corresponded to readmissions, of which 28% were admitted once and 67.6% hospitalized one to four times [28] and in a 12-month follow-up or intervention studies, a survey comparing a group of patients with voluntary hospitalization and a group with involuntary hospitalization found that 37% and 27% of the participants, respectively, were readmitted once during the period [29]. Major differences with respect to our work present a study using a three-year criterion, shows that 55.6% of the participants were readmitted [30]

Numerous studies have examined the relation between the medico-psychiatric comorbidities and economic outcomes for the health. The majority of studies have examined the impact of comorbidities in three measurements of results: length of admission, medical expenditure and rates of readmission. These parameters are considered to be the main generators of expenditure in health care.

Wolf et al. [31] show that patients with a psychiatric comorbidity have a 40% higher cost. Their average episode had a cost €1344 higher than an average episode without psychiatric comorbidities with the same principal diagnosis and after controlling other comorbidities, age and sex. Reimbursements were €1004 higher (28%) and were associated with an increase in daily costs and reimbursements of €207 and €151, respectively. The psychiatric comorbidities were associated with an increase in the cost per episode in somatic hospital care.

Another British study [32] estimated hospital expenditure on psychiatric patients to be £1717 on average, although the sample for this study was the totality of the population with psychiatric diagnoses registered in the Clinical Practice Research Datalink (CPRD), regardless of whether or not they had been admitted to hospital. According to this study, age and number of comorbidities were key factors in influencing a greater hospital expenditure.

Obviously, our work is not without limitations, first, the information on DRGs and resource consumption is obtained with the discharge of patients, who may have been subject to “internal transfers” within the hospital to another clinical service as a consequence of morbidity, in which case the resource consumption of the psychiatry service where they were first admitted is not recorded. Another limitation is the lack of information on the type of comorbidity for each patient as well as the reference of the type of substance abuse per patient. Likewise, the use of ICD9CM codes does not allow us to capture the degree of severity of the illness per patient. Moreover, on occasions, all the diagnoses of the patients are not registered with the ICD9, so the comorbidity of some patients may not be correctly represented, so this should be considered as a limitation of this study.

## 5. Conclusion

The group of schizophrenic patients presents a higher average number of stays and hospital expenditure. With psychiatric patients, neither age nor sex were variables that helped to explain hospital expenditure, nor were the majority of comorbidities. This is in contrast with the majority of medical hospitalisation services due to the particular characteristics of mental health.

## Data Availability

Data is available upon request to authors.

## Notes

1 Indicates that the patient is a smoker at the time of admission.

2 293.0 Delirium due to diseases classified elsewhere. Acute: confusional state infectious psychosis post-traumatic organic psychosis organic reaction psycho-organic syndrome Epileptic: confusional state twilight state Acute psychosis associated with endocrine, metabolic or cerebrovascular disorder. 293.1 Subacute Delirium: state of mental confusion post-traumatic infectious psychosis, psychosis associated with endocrine disorder or metabolic organic reaction (psycho-organic syndrome). Illnesses caused by drugs that are coded as 291.0 to 291.9 –by alcohol-, and 292.82 - other drugs- are excluded.

## Annexe I

**Table:**

Coefficients^a^						
Model		Non standardized coefficients		Standardized coefficients	t	Sig.
		Beta	Dev. error	Beta		
1	(Constant)	9234	0.022		423,029	0.000
	Gender	−0.002	0.009	−0.002	−0.226	0.821
	Substance abuse	0.036	0.010	0.034	3.748	0.000
	ConTP	−0.027	0.015	−0.017	−1.877	0.061
	Hospital readmission	−0.034	0.013	−0.024	−2.735	0.006
	Chapter 1 ICD	0.014	0.016	0.009	0.914	0.361
	Chapter 2 ICD	0.043	0.027	0.014	1.564	0.118
	Chapter 3 ICD	0.013	0.012	0.012	1.089	0.276
	Chapter 4 ICD	0.027	0.022	0.011	1.247	0.212
	Chapter 5 ICD	0.005	0.377	0.005	0.013	0.990
	Chapter 6 ICD	0.015	0.377	0.016	0.041	0.968
	Chapter 7 ICD	−0.038	0.014	−0.032	−2.756	0.006
	Chapter 8 ICD	−0.029	0.019	−0.014	−1.519	0.129
	Chapter 9 ICD	−0.011	0.017	−0.006	−0.652	0.515
	Chapter 11 ICD	0.089	0.218	0.004	0.411	0.681
	Chapter 12 ICD	−0.014	0.027	−0.005	−0.518	0.604
	Chapter 13 ICD	0.006	0.018	0.003	0.353	0.724
	Chapter 14 ICD	0.025	0.033	0.007	0.739	0.460
	Chapter 15 ICD	−0.049	0.143	−0.003	−0.343	0.731
	Chapter 16 ICD	−0.015	0.017	−0.008	−0.875	0.382
	Chapter 17 ICD	0.058	0.026	0.026	2.268	0.023
	Chapter 18 ICD	0.012	0.011	0.012	1.109	0.268
	Chapter 19 ICD	0.008	0.023	0.004	0.338	0.735
	1 psychiatric comorbidity	−0.006	0.012	−0.004	−0.459	0.646
	2 psychiatric comorbidity	−0.025	0.015	−0.016	−1.712	0.087
	3 psychiatric comorbidity	−0.007	0.012	−0.007	−0.610	0.542
	Cost group 2	−0.694	0.013	−0.529	−54.190	0.000
	Cost group 3	−0.578	0.010	−0.543	−56.527	0.000
	1 comorbidity	0.035	0.017	0.026	2.049	0.041
	2 comorbidities	0.000	0.019	0.000	−0.006	0.995
	3 comorbidities	0.002	0.021	0.002	0.113	0.910
	4 comorbidities	−0.026	0.024	−0.016	−1.107	0.268
	5 comorbidities	0.008	0.026	0.004	0.309	0.758
	6 or more comorbidities	0.004	0.029	0.003	0.144	0.885

^a^Variable dependent: LNCOST.
